# Neuroinflammation and white matter microstructure as mediators of cognitive deficits in offspring of parents with bipolar disorder

**DOI:** 10.1093/braincomms/fcag181

**Published:** 2026-05-21

**Authors:** Xiaoyue Li, Wenjin Zou, Robin Shao, Weicong Lu, Lishuo Chao, Xuanhao Zhao, Kuan-Pin Su, Kangguang Lin

**Affiliations:** Department of Affective Disorder, The Affiliated Brain Hospital, Guangzhou Medical University, Guangzhou 510145, Guangdong, P.R. China; Key Laboratory of Neurogenetics and Channelopathies of Guangdong Province and the Ministry of Education of China, Guangzhou Medical University, Guangzhou 510145, Guangdong, P.R. China; Department of Affective Disorder, The Affiliated Brain Hospital, Guangzhou Medical University, Guangzhou 510145, Guangdong, P.R. China; Key Laboratory of Neurogenetics and Channelopathies of Guangdong Province and the Ministry of Education of China, Guangzhou Medical University, Guangzhou 510145, Guangdong, P.R. China; Department of Psychology, The University of Hong Kong, Hong Kong 000000, P.R. China; Department of Affective Disorder, The Affiliated Brain Hospital, Guangzhou Medical University, Guangzhou 510145, Guangdong, P.R. China; Key Laboratory of Neurogenetics and Channelopathies of Guangdong Province and the Ministry of Education of China, Guangzhou Medical University, Guangzhou 510145, Guangdong, P.R. China; Department of Affective Disorder, The Affiliated Brain Hospital, Guangzhou Medical University, Guangzhou 510145, Guangdong, P.R. China; Key Laboratory of Neurogenetics and Channelopathies of Guangdong Province and the Ministry of Education of China, Guangzhou Medical University, Guangzhou 510145, Guangdong, P.R. China; Department of Affective Disorder, The Affiliated Brain Hospital, Guangzhou Medical University, Guangzhou 510145, Guangdong, P.R. China; Key Laboratory of Neurogenetics and Channelopathies of Guangdong Province and the Ministry of Education of China, Guangzhou Medical University, Guangzhou 510145, Guangdong, P.R. China; Department of Psychiatry and Neuroscience, College of Medicine, China Medical University, Taichung 406040, Taiwan; Department of Affective Disorder, The Affiliated Brain Hospital, Guangzhou Medical University, Guangzhou 510145, Guangdong, P.R. China; Key Laboratory of Neurogenetics and Channelopathies of Guangdong Province and the Ministry of Education of China, Guangzhou Medical University, Guangzhou 510145, Guangdong, P.R. China; Department of Psychiatry and Behavioral Sciences, School of Medicine, The Chinese University of Hong Kong, Shenzhen (CUHK-Shenzhen), Shenzhen ‌518172, Guangdong, P.R. China; Department of Neurology, Lecong Hospital of Shunde, Foshan 528315, Guangdong, P.R. China; The Third Affiliated Hospital of Henan Medical University, Xinxiang 453000, Henan, P.R. China

**Keywords:** bipolar disorder, neuroinflammation, white matter, familial risk, information processing speed

## Abstract

Neuroinflammation and white matter disruptions have been implicated in cognitive impairments associated with bipolar disorder, particularly in information processing speed (IPS). These neurological factors may manifest in at-risk offspring, even prior to the full development of the disorder, emphasizing the importance of investigating their role in early cognitive changes associated with bipolar disorder. This study examines these associations in offspring with varying levels of risk for bipolar disorder. Offspring of parents with bipolar disorder were classified as asymptomatic (AO, *n* = 41) or symptomatic (SO, *n* = 35) groups and age-matched healthy controls (HCs, *n* = 32). We assessed serum interleukin-6 (IL-6) levels, IPS performance and fractional anisotropy (FA) of the forceps major (FM) connecting the occipital lobes. Compared to AO, SO demonstrated significantly higher IL-6 levels, reduced FM FA and lower IPS performance, while the AO group exhibited preserved FM FA and IPS performance comparable to HCs. Serial mediation analysis revealed that symptomatic status among offspring at familial risk exerted a significant total indirect effect (*β* = −3.83, 95% CI [−7.28, −0.84]) on IPS performance through the pathways involving IL-6 and FM FA. This indirect effect accounted for 42.46% of the total effect, indicating significant full mediation. Our findings suggest that neuroinflammation and FM microstructure mediate the association between symptomatic status for bipolar disorder offspring and IPS deficits in offspring. This highlights potential neural mechanisms underlying cognitive dysfunction in pre-symptomatic and symptomatic stages of bipolar disorder.

## Teaser

Li *et al*. report that increased inflammation and altered white matter structure are linked to slower information processing in young people at familial risk for bipolar disorder. This study identifies a potential brain mechanism for early cognitive changes, highlighting targets for early intervention.

## Graphical Abstract

**Graphical Abstract:**
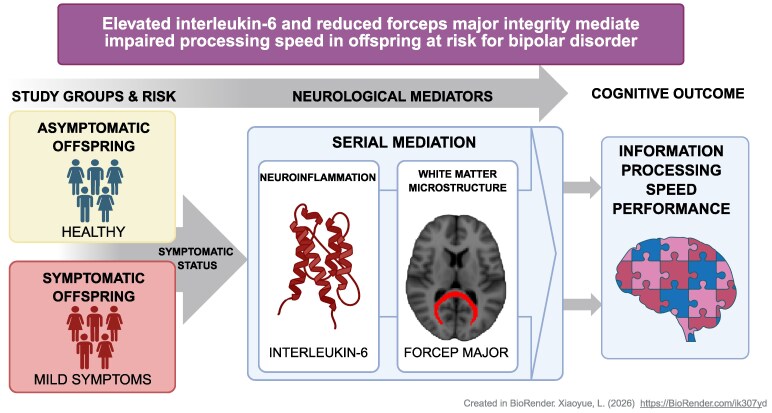

## Introduction

Bipolar disorder is a severe mental illness characterized by a significant hereditary component.^1,2^ Offspring with a family history of bipolar disorder are considered at risk, while those with both a family history and subthreshold symptoms are classified as ultra-high risk.^3,4^ Studies suggest that roughly 25% of at-risk individuals may develop the disorder.^5,6^ Our prior research and others have shown that bipolar disorder offspring exhibit variable cognitive impairments, with distinct profiles depending on whether they are symptomatic or asymptomatic.^7,8^ Information processing speed (IPS) is a fundamental cognitive function reflecting the ability to quickly and efficiently process information and generate responses.^9,10^ Slower IPS can negatively impact performance on higher-order cognitive tasks requiring complex processing, such as working memory and executive function.^11,12^ Individuals with bipolar disorder exhibit a steeper decline in IPS with age compared to healthy controls (HCs), even during symptom remission.^13^ Furthermore, research suggests IPS abnormalities are prevalent in bipolar disorder offspring.^7^ Higher IPS is associated with better outcomes in bipolar disorder, including remission, occupational status and life goals.^14^

The exact mechanisms behind IPS impairments in bipolar disorder and offspring remain unclear. Deficits in white matter (WM) microstructure have been linked to IPS impairments.^7,13,15-17^ Specific WM abnormality within the corpus callosum (CC), which connects the prefrontal cortex, can disrupt IPS by hindering information transmission.^18-20^ Additionally, WM microstructure in the cingulum and corona radiata is linked to IPS.^21,22^ The occipital lobe also plays a crucial role in IPS, influencing visual perception, object recognition, spatial processing and visual memory.^23^ Research suggests structural changes and disrupted connectivity within the occipital lobe occur in bipolar disorder patients.^24,25^ However, the impact of WM alterations, especially those linked to the occipital lobe, on IPS, particularly before the complete onset of bipolar disorder, is under-explored. This highlights the need for further investigation.

In recent decades, systemic inflammation has been strongly linked to cognitive impairment, especially in bipolar disorder.^26^ Persistent low-level inflammation, which intensifies during acute mood episodes, may accelerate cognitive decline in bipolar disorder patients.^27^ Genetic studies have identified a unique inflammatory gene expression in bipolar disorder patients and their offspring, forming a ‘genetic signature’ preceding clinical onset.^28^ Interleukin-6 (IL-6), a principal pro-inflammatory cytokine, is a significant focus area. Research consistently shows elevated IL-6 levels in bipolar disorder patients, persisting across mood states.^29^ Studies in pregnant mice suggest that transient IL-6 elevations can alter brain connectivity in offspring, impacting neural pathways crucial for cognitive function.^30^ Our previous research revealed higher serum IL-6 levels in symptomatic bipolar disorder offspring, associated with reduced grey matter volume in the rostral anterior cingulate cortex and impaired attention, indicating the presence of distinct neurocognitive-inflammatory profiles in two subgroup ‘unaffected’ bipolar disorder offspring.^31^ However, the interactions between IL-6, WM and IPS, and whether they undergo stage-specific changes in these two subgroups of offspring, remain unknown.

Considering the points mentioned, this research is motivated by the hypothesis that inflammation-induced changes in WM could significantly hinder the transmission of information, thus impairing cognitive efficiency. This study specifically examines four crucial WM tracts connected to the occipital lobe: the inferior fronto-occipital fasciculus (IFOF), the inferior longitudinal fasciculus (ILF), the superior longitudinal fasciculus (SLF) and the forceps major (FM). The choice of these tracts was strategic, based on their essential roles in the neural framework supporting IPS. The IFOF is critical for merging visual stimuli with higher cognitive functions by linking the frontal and occipital lobes.^32^ The ILF is central to the processing of visual memories and recognition, bridging the occipital and temporal lobes.^33,34^ The SLF is key in coordinating visual, spatial and executive functions across the occipital, parietal and frontal lobes, emphasizing its significance in complex visual tasks.^35,36^ Furthermore, the FM, part of the CC, is vital for the bilateral coordination of visual information, facilitating a unified visual perception across both hemispheres.^37,38^

This study aimed to assess the relationship between IL-6 levels, the regions of interest (ROIs) of the WM tracts and IPS in the familial at-risk offspring of individuals with bipolar disorder. Specifically, it sought to determine whether symptomatic and asymptomatic individuals exhibit distinct inflammation-brain-cognition profiles in IPS, which could serve as biomarkers for resilience to or risk for bipolar disorder. We examined the following hypotheses: (i) IL-6 levels would correlate with the ROIs’ microstructure; (ii) the ROIs’ microstructure would be associated with participants’ IPS; and (iii) IL-6 levels and ROIs’ microstructure may mediate varying effects on IPS among asymptomatic and symptomatic bipolar disorder offspring, reflecting their distinct aetiological profiles.

## Materials and methods

### Participants

The dataset was derived from the Recognition and Early Intervention on Prodromal Bipolar Disorder (REI-PBD) study, initiated in 2013, focusing on tracking a cohort of offspring of parents with bipolar disorder.^39^ This study was approved by the Institutional Review Board of The Affiliated Brain Hospital of Guangzhou Medical University. Written informed consent was obtained from all participants and their guardians (if under the age of 18).

Participants were assessed for lifetime Axis I disorders using either the Schedule for Affective Disorders and Schizophrenia for School-aged Children−Present and Lifetime versions (K-SADS-PL) or, for individuals over 18 years, the Structured Clinical Interview for DSM-IV-TR Axis I Disorders, Research Version, Patient Edition (SCID-I/P). The criteria for participant inclusion were as follows: (i) presence of a biological parent diagnosed with bipolar disorder; (ii) participants aged between 8 and 28 years; and (iii) individuals of Han ethnicity with Chinese heritage. Depending on the manifestation of specific subthreshold mood syndromes, participants were categorized into symptomatic offspring (SO) or asymptomatic offspring (AO). The subthreshold syndromes included the following: (i) experiencing two or three hypomanic symptoms for a minimum of 4 days, not meeting the hypomania criteria; (ii) exhibiting two or more symptoms indicative of a major depressive episode for at least 1 week, without meeting the major depressive episode criteria in terms of symptom count or duration of 2 weeks; and (iii) displaying one or more attenuated psychotic symptoms, each lasting a minimum of 10 min, occurring 2–7 times weekly for a span exceeding 3 months.^40^ Conversely, AO did not exhibit the aforementioned subthreshold mood symptoms and remained devoid of any DSM-IV–defined psychiatric disorder. Exclusion criteria comprised current or past DSM-IV-TR Axis I disorders; substance or alcohol abuse, pregnancy, hypo-/hyperthyroidism and traumatic brain injuries; or any impediment preventing completion of MR scanning or resulting in artefact-laden MR images. In total, 35 SO and 41 AO were enrolled in this study. Age-matched HC participants were recruited through self-referral, targeted advertisements or community referrals, utilizing identical recruitment methodologies as those implemented for the offspring of parents with bipolar disorder.

### Peripheral IL-6 level measurements

Participants’ peripheral blood samples were collected from upper limb veins at The Affiliated Brain Hospital of Guangzhou Medical University, China, within the time frame of 8:00 a.m. to 10:00 a.m. Each sample, amounting to 5 mL, was deposited into vacutainer tubes devoid of additives, followed by centrifugation to extract the respective sera. These sera samples were then meticulously stored at −80°C for subsequent evaluations. Utilizing a bead-based human ProcartaPlex immunoassay (Thermo Fisher Scientific, Waltham, MA, USA), the concentration of IL-6 was ascertained, strictly following the manufacturer’s stipulated protocols. Initially, antibody-coated beads underwent a 120-min incubation, either with premixed standards or sample supernatants, at room temperature, rotating at a consistent speed of 500 r/min via a 96-well plate mixer (Olabo, Jinan, Shandong Province). Subsequent steps involved introducing a blend of detection antibodies, followed by a 30-min incubation under the aforementioned conditions. Post-incubation, a sequence of washing and the introduction of phycoerythrin-conjugated streptavidin ensued, all under consistent incubation parameters. Ultimately, the resuspended beads in Bio-Plex cytokine assay buffer (Bio-Rad, San Francisco, CA, USA) were subjected to analysis using the Bio-Plex 200 system, configured with an optimized low photomultiplier tube setting. Data interpretation was facilitated using the ProcartaPlex Analyst 1.0 software (Thermo Fisher Scientific).^41,42^ The reliable detection range for IL-6, defined by the lower and upper limits of quantitation, was 0.38–6244 pg/mL. The average intra-assay coefficient of variation was 2.82%, and no sample was below the assay detection limit.

### MRI acquisition and preprocessing

MRI data for participants were obtained using a Philips 3.0 T MRI scanner, which was equipped with an eight-channel SENSE head-coil. A series of 32 non-collinear diffusion images (b-value = 1000 s/mm^2) and a reference image with no diffusion weighting (b0) were gathered employing an echo-planar imaging sequence. The specific imaging parameters incorporated a field of view set at 256 × 256, a repetition time (TR)/echo time (TE) of 10086/91 ms, a consistent slice thickness of 2 mm without intervals, a matrix size of 128 × 128 and a voxel dimension of 2 × 2 × 2 mm^3^. To correct for potential eddy current distortions and any head motion artefacts, a registration process aligned the diffusion-weighted images to the b0 reference image. Subsequent adjustments to the b-matrix rotations were executed utilizing the FMRIB Diffusion Toolbox from the FMRIB Software Library (FSL). Ultimately, the fractional anisotropy (FA) was determined employing the DTIfit application, as detailed on the FSL platform (https://fsl.fmrib.ox.ac.uk/fsl/fslwiki/FDT). Tract region of interests (ROIs), including IFOF, ILF, SLF and FM, were created from probabilistic tract map based on JHU ICBM-DTI-81 atlas using ‘fslmaths’ command within FSL. Mean FA of tract ROI were calculated for each participant.^43^

### Clinical and visual processing speed measure

The Hamilton Depression Rating Scale (HAMD), the Brief Psychiatric Rating Scale (BPRS) and the Young Mania Rating Scale (YMRS) were applied to assess the severity of depression, psychotic symptoms and mania, respectively.^44^ IPS was assessed through the symbol coding task of the Brief Assessment of Cognition in Schizophrenia (BACS SC),^45^ a subscale derived from The MATRICS Consensus Cognitive Battery.^46^ During this assessment, participants are instructed to match nonmeaningful symbols with numerals 1–9 on a response sheet within a 90-s time frame, using a provided key as reference.

### Statistical analysis

We defined univariate outliers as data points exceeding 3.5 SD from the mean and excluded them from the analysis. IL-6 levels were log_10_ transformed to normalize their distribution. Demographics and clinical variables were analysed using univariate ANOVA or non-parametric tests (Kruskal–Wallis test and chi-square test) according to types of variables and data distribution. Between-group comparisons on IL-6 levels, FA of tract ROIs and BACS SC scores were analysed using general linear models that separately included those variables as the dependent variable, groups as a fixed factor and age (squared) and sex as covariates. Bonferroni correction was applied to adjust for multiple comparisons. FA of tract ROIs were subjected to false discovery rate (FDR) correction to control for multiple comparisons, ensuring the robustness and reliability of the subsequent analyses. Partial correlation analyses were conducted between the FA of tract ROIs and the IL-6 level, between the FA of tract ROIs and the BACS SC score and between the IL-6 level and BACS SC score, controlling for age (squared) and sex. To examine the serial mediation effects of IL-6 levels and FA values within tract ROIs linking the binary familial risk group variable to BACS SC scores, we conducted mediation analysis for categorical independent variables using Hayes’ PROCESS macro (Model 6) in SPSS. The analysis employed bias-corrected bootstrapping with 5000 resamples to estimate 95% confidence intervals. All inferences were based on two-tailed tests with α = 0.05. Statistical analyses were conducted using SPSS (version 24.0) and R (version 4.1.2).

## Results

### Demographic and clinical characteristics

As shown in Table 1, this study included a total of 76 bipolar disorder offspring (41 categorized as AO and 35 as SO) and 32 HCs. The three groups (AO, SO, and HCs) were matched on age, sex and education. Non-parametric analyses showed significant intergroup differences in HAMD, YMRS and BPRS scores (*P* < 0.05).

**Table 1:** Demographic and clinical data between-group comparison

	AO (*N* = 41)	SO (*N* = 35)	HCs (*N* = 32)		
	M ± SD	M ± SD	M ± SD	Statistic	*P*-value
Demographic information					
Age	17.73 ± 5.68	18.69 ± 6.68	16.32 ± 3.5	1.63^a^	0.2
Sex (female)	24	18	20	0.51^b^	0.78
Education	9.78 ± 3.78	9.17 ± 4.55	9.88 ± 3.15	0.35^a^	0.71
Clinical measure					
HAMD	0.44 ± 0.9	8.4 ± 9.31	0.38 ± 1.05	51.14^c^	<0.01
YMRS	0.59 ± 1.52	1.89 ± 2.34	0.03 ± 0.17	26.39^c^	<0.01
BPRS	18.27 ± 0.63	22.91 ± 5.07	18.24 ± 0.78	54.31^c^	<0.01

Demographic variables were analysed using univariate ANOVA when data were normally distributed (a), the chi-square test for the binary variable of sex (b) and the non-parametric Kruskal–Wallis test when data were not normally distributed (c).

AO, asymptomatic offspring; SO, symptomatic offspring; HCs, healthy controls; HAMD, Hamilton Depression Rating Scale; YMRS, Young Mania Rating Scale; BPRS, Brief Psychiatric Rating Scale; M, mean; SD, standard deviation.

### IL-6 levels and BACS SC score comparisons between groups

Following adjustment for age (squared) and sex, IL-6 levels were significantly higher in the SO group compared to the AO group (*F* = 8.69; *P* < 0.01). The HC group was excluded from IL-6 level analyses due to incomplete data collection. In contrast, significant differences in the BACS SC scores were observed across all three groups (SO, AO, and HCs) after covariate adjustment (*F* = 10.23; *P* < 0.01). *Post hoc* analyses indicated a hierarchical pattern: the AO group scored higher than the SO group (*P* < 0.01), while the HC group exhibited the higher scores relative to the SO group (*P* < 0.01) (Table 2).

**Table 2:** The between-group comparison of IL-6 levels and BACS SC

	AO (*N* = 41)	SO (*N* = 35)	HCs (*N* = 32)			
Variable	M ± SE	M ± SE	M ± SD	*F*-value	*P*-value	*pos* comparisons
IL-6 (log_10_)	0.87 ± 0.01	0.93 ± 0.02	-	8.69	<0.01	AO < SO
BACS SC	61.5 ± 1.72	52.35 ± 1.88	63.81 ± 1.92	10.23	<0.01	AO > SO, HC > SO

IL-6 levels were log_10_ transformed prior to analysis to normalize their distribution. General linear models compared IL-6 levels, and BACS SC scores between groups, with age-squared and sex as covariates.

AO, asymptomatic offspring; SO, symptomatic offspring; HCs, healthy controls; IL-6, interleukin-6; BACS SC, symbol coding of the Brief Assessment of Cognition in Schizophrenia.

### Comparisons of ROI white matter tracts between groups

**Figure 1:**
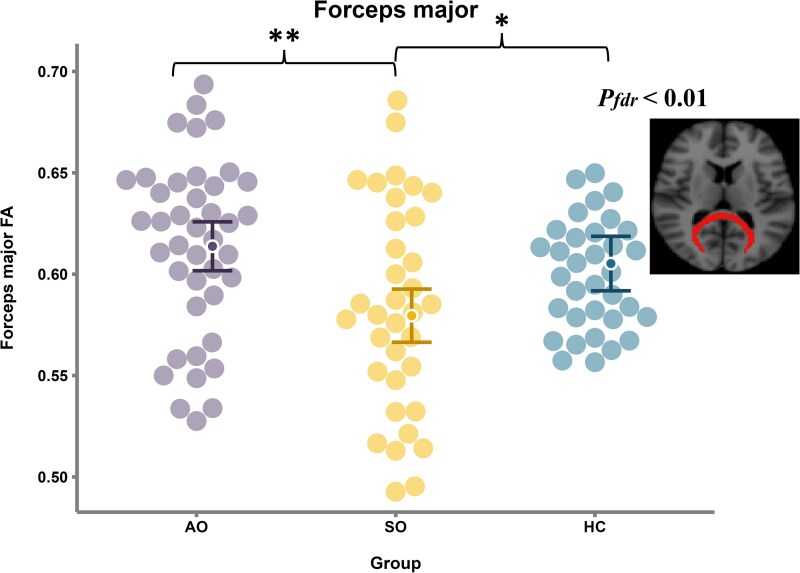
**The between-group comparison of FA of FM.** General linear models were used to compare FA of FM between groups (AO, *n* = 41; SO, *n* = 35; HCs, *n* = 32), with age (squared) and sex as covariates. FA of tract ROIs underwent FDR correction to control for multiple comparisons. Significance levels are indicated as * *P* < 0.05 and ** *P* < 0.01. Each dot is a participant’s FA value. Dots inside the error bars (shown in distinct colors for the three groups) indicate estimated marginal means [adjusted for gender and age (squared)], and error bars represent 95% confidence intervals of those adjusted means. ROIs, regions of interest; FA, fractional anisotropy; AO, asymptomatic offspring; SO, symptomatic offspring; HCs, healthy controls; *Pfdr*, *P*-value after false discovery rate correction.

As demonstrated in Fig. 1, analysis of covariance adjusting for age (squared) and sex revealed significant between-group differences in FM FA across the three groups (*F* = 7.6; *Pfdr* < 0.01). *Post hoc* testing identified a distinct hierarchy: the SO group exhibited reduced FM FA relative to both the AO group (mean difference = 0.03; *P* < 0.01) and HC group (mean difference = −0.03; *P* < 0.01). No between-group differences were observed in IFOF FA, ILF FA or SLF FA (*Pfdr* > 0.05), as shown in Fig. S1 (see the Supplementary material).

### Correlations among FA, IL-6 level and BACS SC score

After controlling for age (squared) and sex, as shown in Fig. S2, the FM FA showed a significant negative correlation with IL-6 level (r = −0.39; *P* < 0.01). Furthermore, the FM FA demonstrated a significant positive correlation with the BACS SC score (r = 0.49; *P* < 0.01). No significant association was observed between the IL-6 level and the BACS SC scores (*P* > 0.05).

### Mediation effect

**Figure 2:**
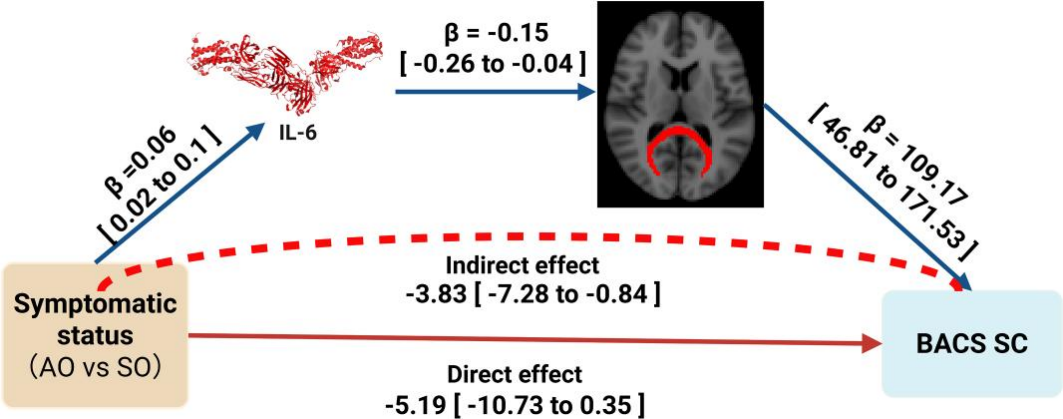
**Model of multiple mediation pathways.** Serial mediation analyses were performed to examine whether inflammatory markers and WM microstructure mediate the relationship between symptomatic status in bipolar disorder offspring and cognitive performance. The figure illustrates the pathway through which differential familial risk (AO, *n* = 41; SO, *n* = 35) influences cognition via IL-6 levels and FM FA. This figure was created with BioRender (https://BioRender.com/fokpk7h, agreement number: EK29DKWBO4).

To examine the potential mechanisms linking symptomatic status in bipolar disorder offspring to cognitive performance through inflammatory markers and WM microstructure, serial mediation analyses were conducted. Table S1 (see the Supplementary material for the full mediation model) shows the mediation analysis results, and Fig. 2 shows the mediation pathway model. These analyses revealed that symptomatic status (AO versus SO) significantly predicted elevated IL-6 levels (β = 0.06, 95% CI [0.02, 0.1]; R^2^ = 0.12; Model 1). Model 2 identified both elevated IL-6 (β = −0.15, 95% CI [−0.26, −0.04]) and symptomatic status (β = −0.03, 95% CI [−0.05, −0.01]) as independent negative predictors of FM FA (R^2^ = 0.34). The final model demonstrated that FM FA was the principal predictor of BACS SC scores (β = 109.17, 95% CI [46.81, 171.53]), with non-significant direct effects for symptomatic status (β = −5.19, 95% CI [−10.73, 0.35]) or IL-6 (β = −0.16, 95% CI [−30.94, 30.62]; R^2^ = 0.4) indicating full mediation through the proposed pathways.

As demonstrated in Table S2 (see the Supplementary material), decomposition of effects revealed a significant total effect of symptomatic status on BACS SC scores (β = −9.02, 95% CI [−14.4, −3.64]) and a non-significant direct effect (β = −5.19, 95% CI [−10.73, 0.35]). The total indirect effect was significant (β = −3.83, 95% CI [−7.28, −0.84]), accounting for 42.46% of the total effect. The pathway through FM FA alone (symptomatic status → FM FA → BACS SC) contributed substantially (β = −2.85, 95% CI [−6.14, −0.37]), while the serial pathway via IL-6 and FM FA (symptomatic status → IL-6 → FM FA → BACS) showed a smaller effect (β = −0.97, 95% CI [−2.39, −0.15]).

## Discussion

To our knowledge, this study is the first to explore the interplay between IL-6 levels, occipital-linked WM microstructure and IPS in bipolar disorder offspring at varying clinical stages. Our findings demonstrate that the SO group showed elevated IL-6, reduced FM FA and poorer IPS performance versus the AO group, while the AO group exhibited preserved FM FA and IPS performance comparable to HCs. Serial mediation analyses revealed that FM FA fully mediated the relationship between symptomatic status in bipolar disorder offspring and a smaller indirect effect via IL-6. This suggests that FM microstructural disruption is the central pathway linking symptom manifestation to cognitive decline, where IL-6 acts secondarily through WM damage rather than direct effects.

It is believed that peripheral immune activation plays a role in the neuroprogression of bipolar disorder.^26^ Studies of unmedicated patients with bipolar disorder have shown that elevated IL-6 levels are associated with reduced functional connectivity in corticolimbic networks implicated in affective processes.^47,48^ Consistent with our hypothesis, we observed elevated IL-6 correlating with reduced FM FA, suggesting peripheral inflammation may associate with structural changes. Pro-inflammatory cytokines, such as IL-6, compromise blood–brain barrier integrity,^49^ permitting immune cell infiltration that activates microglia and reduces oligodendrocyte function—a process crucial for myelination.^50,51^ Such changes adversely affect the WM microstructure. Moreover, cytokines, such as IL-6, stimulate the production of C-reactive protein, which in turn triggers apoptosis by activating pro-apoptotic proteins, including IL-1 subtypes and TNF-α.^52,53^ This process contributes to further impairment of the structure and function of brain.

Critically, IL-6 showed no direct effect on IPS but operated exclusively through FM FA disruption. The FM, located in the splenium of the CC, is vital for regulating IPS and visual attention by enhancing interhemispheric communication between the occipital lobe and superior parietal lobules.^37,54^ Poor myelination along the FM can intricately alter interhemispheric neural communication, potentially slowing neural transmission.^55^ Disruption within the splenium may hinder efficient synchronization of frontoparietal networks between hemispheres, impacting the speed of visual information transmission and allocation of processing resources necessary for visual object recognition and discrimination.^38,54^ While reduced FM FA is a transdiagnostic feature of affective disorders,^56^ our study specifies its role as the final pathway through which inflammation and symptom progression converge in high-risk bipolar disorder offspring. In the clinical context of bipolar disorder, our findings highlight the sensitivity and vulnerability of the FM to inflammatory environments. This underscores how these vulnerabilities may predispose SO to cognitive deficits and the onset of the illness.^4^

In our study, we found no differences in the ILF, IFOF and SLF between AO and SO groups, except for the FM. Priorreports have shown widespread WM alterations in bipolar disorder offspring.^57-59^ However, there has been limited investigation into the differences between symptomatic and asymptomatic individuals within bipolar disorder offspring. Notably, our findings contrast with the increased FA reported by another study in bipolar disorder offspring compared to HCs.^59^ Two reasons may explain the diverging findings of decreased FA in bipolar disorder offspring as observed in our study, but increased FA in bipolar disorder offspring as reported by the previous study.^59^ First, a majority of participants in the previous study^59^ were receiving medications, which could boost their WM integrity,^60^ while none of our participants were on medication. Following this rationale, decreased FM FA may be considered as a risk marker, which is improved by medication. Second, the bipolar offspring in the previous study^59^ had no mania history, while our SO already manifested subthreshold mood symptoms. This would also suggest that the decreased FM FA is a vulnerability marker among individuals with both familial and symptomatic risks of bipolar disorder, while increased FM FA might be a resilience marker among unaffected bipolar disorder offspring. Additionally, Nery *et al*.^61^ found no differences in WM volumes among symptomatic bipolar disorder offspring, asymptomatic bipolar disorder offspring and HCs.^58^ This negative finding was inconsistent with ours, possibly due to the limited sensitivity of morphometry analyses in capturing microstructural properties and spatial organization of WM. FA measures axonal microstructure and fibre alignment, providing insights into WM microstructural changes. Reduced FA can indicate demyelination, axonal damage or alterations in fibre density or orientation.^62^

Several limitations warrant consideration. First, the modest sample size constrains statistical power and may limit generalizability. Second, our whole-tract analytical approach cannot resolve microstructural heterogeneity within WM tracts—particularly functionally distinct SLF subcomponents (SLF I–III)—which may differentially impact IPS.^63^ Third, while our mediation analysis deliberately focused on mean FA from *a priori* regions for group comparisons, this methodology lacks sensitivity for detecting localized WM-cognition associations.^20^ Finally, the identified IL-6 → FM FA → IPS deficit pathway was specifically validated in familial bipolar disorder-risk individuals; its generalizability beyond this population remains unestablished due to absent IL-6 data in HCs, precluding cross-group validation of this mechanistic model.

In conclusion, our study indicates that IL-6 levels may interact with FM microstructure, contributing to IPS differences in both SO and AO of individuals with bipolar disorder. The observed association between FM-connected occipital alterations and clinical profiles suggests a potential neuroanatomical correlate of vulnerability. These cross-sectional findings underscore the need for longitudinal investigations to determine the predictive value of this pathway for illness development.

## Supplementary Material

fcag181_Supplementary_Data

## Data Availability

Data will be made available upon reasonable request. Qualified researchers can submit proposals to klin@cuhk.edu.cn and must sign a data access agreement. Code for analysis is publicly available at https://github.com/lixiaoyue8922490/script.
