# Supplementary material for: Neuroinflammation and white matter microstructure as mediators of cognitive deficits in offspring of parents with bipolar disorder
Source: Brain Commun. 2026 May 21;8(3):fcag181. doi: 10.1093/braincomms/fcag181 (PMC13225265; doi:10.1093/braincomms/fcag181)
Supplement: fcag181_Supplementary_Data [file fcag181_supplementary_data.docx]

**Supplemenraty material**

**Table S1 Results of the multiple mediation analysis**

| **Variable** | **Predictive variable** | ***R^2^*** | ***F*** |  | ***b*** | ***SE*** | ***t*** | ***LLCI*** | ***ULCI*** |
| --- | --- | --- | --- | --- | --- | --- | --- | --- | --- |
| **Model 1** |  | 0.12 | 3.4 |  |  |  |  |  |  |
| IL-6 (Log _10_) | Symptomatic status （AO vs SO） |  |  |  | 0.06 | 0.02 | 2.95 | 0.02 | 0.1 |
| **Model 2** |  | 0.34 | 9.26 |  |  |  |  |  |  |
| FM | Symptomatic status （AO vs SO） |  |  |  | -0.03 | 0.01 | -2.59 | -0.05 | -0.01 |
|  | IL-6 (Log _10_) |  |  |  | -0.15 | 0.06 | -2.68 | -0.26 | -0.04 |
| **Model 3** |  | 0.4 | 9.17 |  |  |  |  |  |  |
| BACS SC | Symptomatic status （AO vs SO） |  |  |  | -5.19 | 2.78 | -1.87 | -10.73 | 0.35 |
|  | IL-6 (Log _10_) |  |  |  | -0.16 | 15.43 | -0.01 | -30.94 | 30.62 |
|  | FM |  |  |  | 109.17 | 31.27 | 3.49 | 46.81 | 171.53 |

After adjusting for age (squared) and sex, mediation analysis was conducted to assess the mediating roles of IL-6 levels and FM FA in the relationship between various symptomatic status (AO vs. SO) and BACS SC scores. AO: Asymptomatic offspring; SO: Symptomatic offspring; IL-6: Interleukin-6; FM: Forceps major; BACS SC: Symbol coding of the Brief Assessment of Cognition in Schizophrenia

**Table S2 Multiple Mediation Effects the familial risk and BACS SC**

|  | ***Effect*** | ***SE*** | ***LLCI*** | ***ULCI*** | **Indirect to total effect** |
| --- | --- | --- | --- | --- | --- |
| **Total effect** | -9.02 | 2.7 | -14.4 | -3.64 | - |
| **Direct effect** | -5.19 | 2.78 | -10.73 | 0.35 | - |
| **Total indirect effect** | -3.83 | 1.65 | -7.28 | -0.84 | 42.46% |
| **Ind 1** | -0.01 | 0.95 | -1.8 | 2.06 | - |
| **Ind 2** | -2.85 | 1.46 | -6.14 | -0.37 | 31.6% |
| **Ind 3** | -0.97 | 0.58 | -2.39 | -0.15 | 10.75% |

**Ind1: Symptomatic status → IL-6 (Log _10_) → BACS SC;**

**Ind2: Symptomatic status → FM → BACS SC;**

**Ind3: Symptomatic status → IL-6 (Log _10_) → FM → BACS SC;**

AO: Asymptomatic offspring; SO: Symptomatic offspring; IL-6: Interleukin-6; FM: Forceps major; BACS SC: Symbol coding of the Brief Assessment of Cognition in Schizophrenia


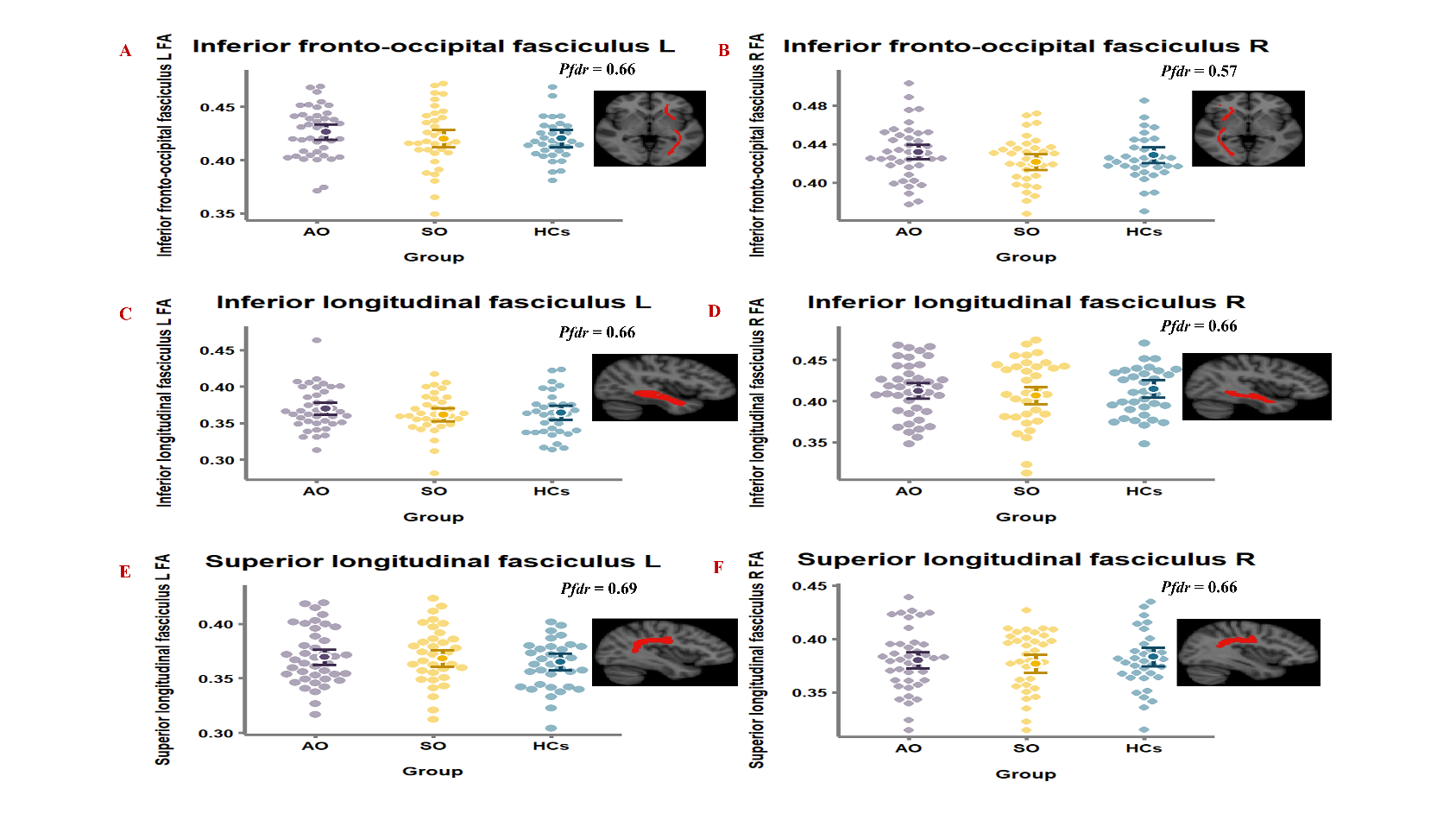
**Figure S1 The between-group comparison of FA of tract ROIs**

General linear models were used to compare FA of tract ROIs between groups (AO, n=41; SO, n=35; HCs, n=32), with age (squared) and sex as covariates. FA of tract ROIs underwent FDR correction to control for multiple comparisons. Each dot represents one participant’s FA value. Colored dots inside the error bars show estimated marginal means (adjusted for gender and age (squared)), and error bars indicate 95% confidence intervals of those adjusted means (A-F). ROIs: Regions of interest; FA: Fractional anisotropy; IFOF: Inferior fronto-occipital fasciculus; ILF: Inferior longitudinal fasciculus; SLF: Superior longitudinal fasciculus; AO: Asymptomatic offspring; SO: Symptomatic offspring; HCs: Health controls

**
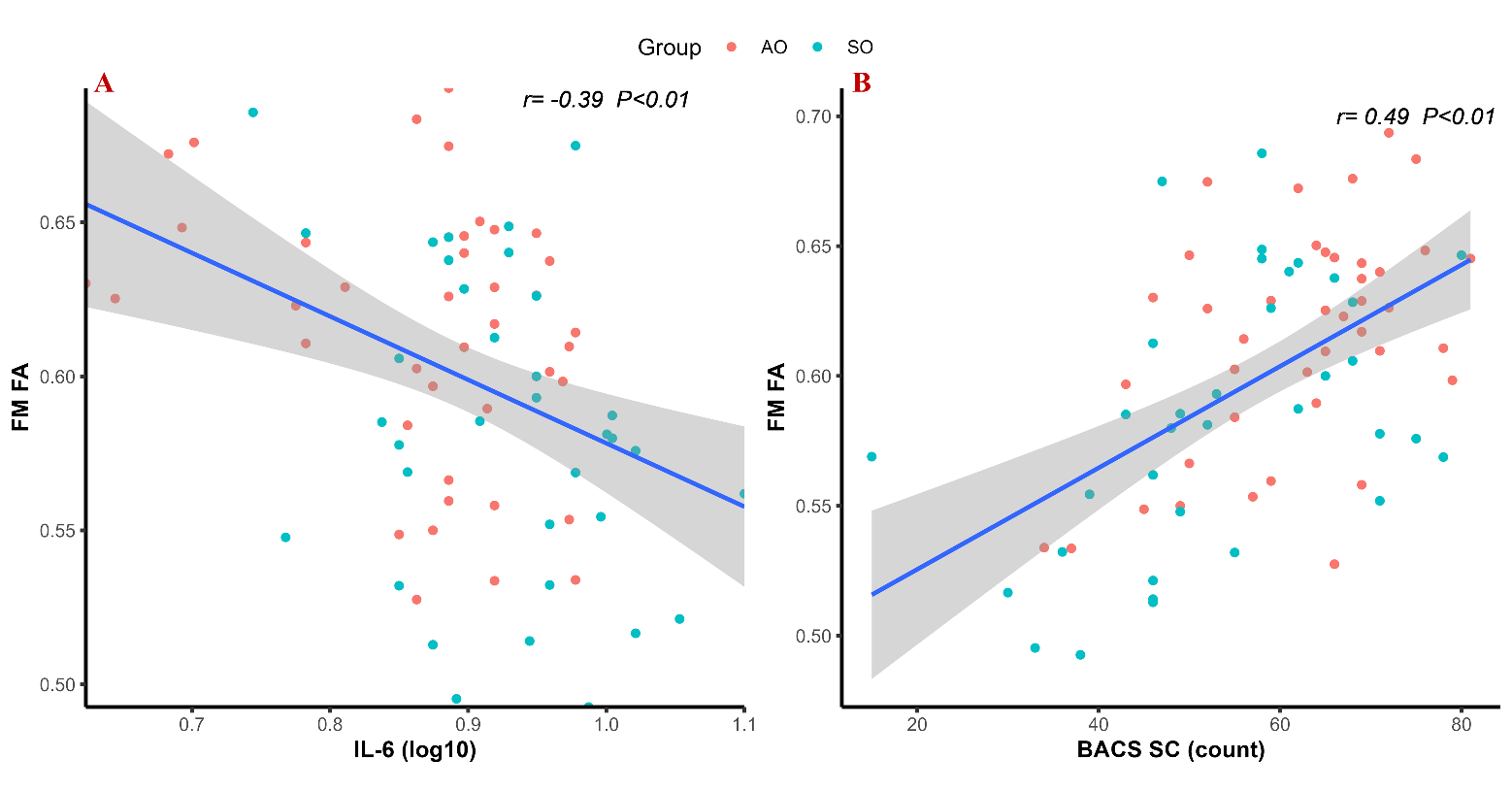
Figure S2 Correlations among FA, IL-6 level and BACS SC score**

Scatter plots showing individual data points for each participant. Partial correlation analyses (adjusting for age (squared) and gender) examining the relationships between significant variations in FM FA and both IL‑6 levels (A) and BACS SC scores (B) in asymptomatic offspring (AO, n=41) and symptomatic offspring (SO, n=35). FA: Fractional anisotropy; AO: Asymptomatic offspring; SO: Symptomatic offspring; IL-6: Interleukin-6; FM: Forceps major; BACS SC: Symbol coding of the Brief Assessment of Cognition in Schizophrenia
